# Retraction: Mobile phone text messaging and Telephone follow-up in type 2 diabetic patients for 3 months: a comparative study

**DOI:** 10.1186/2251-6581-11-21

**Published:** 2013-03-07

**Authors:** Mirta Zolfaghari, Seyedeh A Mousavifar, Hamid Haghani

**Affiliations:** 1 Nursing & Midwifery Care Research Center, Tehran University of Medical Science, Tehran, Iran; 2 School of Nursing and Midwifery, Tehran University of Medical Science, Tehran, Iran; 3 Department of Biostatistics, Tehran University of Medical Sciences, Tehran, Iran

## 

This article [1] has been retracted as it has already been published in the Iranian Journal of Diabetes and Obesity [2].
